# Supplementary material for: Exploring the impact of college graduates’ place attachment on entrepreneurial intention upon returning to hometowns: A study based on the theory of planned behavior
Source: PLoS One. 2024 Mar 29;19(3):e0300312. doi: 10.1371/journal.pone.0300312 (PMC10980218; doi:10.1371/journal.pone.0300312)
Supplement: Attachment — Submitted filename: Response to Reviewer 2 Comments.docx [file pone.0300312.s001.docx]

Response to Reviewer 2 Comments

**Point 1:** Title and abstract – The title is very descriptive but not overly wordy. It needs to go wider for a more specific representation of the study's aims.

**Response 1:** We appreciate your feedback and suggestions about the title. As required, we have made the following revision.

" Exploring the Impact of College Graduates' Place Attachment on Entrepreneurial Intention upon Returning to Hometowns: A Study Based on the Theory of Planned Behavior" (See Lines 4-6)

**Point 2:** Please make your abstract attractive to readers (simple sentences without any repetition) and include 2-3 sentences ready to be cited exactly as they are. In 1 paragraph, your abstract should tell the readers why the study is important (maximum 25% of the text), what you did, i.e. your methodology (maximum 25% of the text), and what you found, i.e. main research results and their major implications (50% of the text). This is very important to promote your work because of the growing trend that authors use Google search to find and cite papers based on the abstract (instead of reading the full paper).

**Response 2:** We are thankful for the reviewer’s comments and suggestions on the abstract. As required, we have the following revisions.

“The issue of the continuing decline of rural areas caused by urbanization has become a global concern. Encouraging college graduates to return to their hometowns to start businesses is an important initiative for countries to achieve sustainable rural development. Drawing from the Theory of Planned Behavior (TPB), this study introduces two additional variables: place attachment and entrepreneurial self-efficacy. Through a series of three model refinements, a comprehensive theoretical framework has been formulated to elucidate Chinese college graduates’ hometown-based entrepreneurial intention and behavior. The samples for this study were 1151 college graduates selected from diverse universities across China. This study aims to explore the influence of their hometown-based entrepreneurial intention using Structural Equation Modelling. This analytical approach illuminates how variables such as college graduates’ place attachment, entrepreneurial self-efficacy, subjective norm for hometown-based entrepreneurship, and attitude towards hometown-based entrepreneurship affected their hometown-based entrepreneurial intention. The research findings reveal the following insights; (1) The overall levels of college graduates’ place attachment and hometown-based entrepreneurial intention were not high. (2) College graduates’ place attachment, entrepreneurial self-efficacy, subjective norm for hometown-based entrepreneurship, and attitude towards hometown-based entrepreneurship, had a positive impact on their hometown-based entrepreneurial intention. (3) College graduates’ place attachment and subjective norm for hometown-based entrepreneurship had a significant impact on their hometown-based entrepreneurial intention through the mediating variable of entrepreneurial self-efficacy. This study then makes policy recommendations from the three aspects including economic inputs, cultural development, and educational practice.” (See Lines 28-49)

**Point 3:** Regarding the Introduction, the first two paragraphs need to be enhanced with more arguments exemplifying the importance of entrepreneurship and this research. There is a lot of published work in 2021 and 2022, such as Barba et al. (2022) https://doi.org/10.1016/j.iedeen.2021.100184 or Calderon-Milan et al. (2020), https://doi.org/10.3390/su12031079, to strengthen the narrative on this.

**Response 3:** We are grateful for the reviewer’s comments and suggestions about this. As required, we have made the following revision.

“…… Entrepreneurial activity is recognized as a vital endeavor that merits encouragement due to its significant contributions to the economic and social development of a given area. It serves as a means of creating employment opportunities and plays an essential role in addressing this issue [3]. For example, China has been steadfast in its pursuit of the 'rural revitalization' strategy since 2017. The primary objective of this strategy is to rectify the growing imbalance between rural and urban areas, ultimately striving for more sustainable development. A pivotal approach is to attract both the population and other factors towards rural regions [4]. Consequently, promoting the return of college graduates to their hometowns to initiate businesses emerges as a crucial initiative for nations seeking sustainable rural development.” (See Lines 54-63)

"The promotion of entrepreneurial behavior stands as a significant matter of shared concern among governments and scholars across the globe [11]. The academic community has conducted a substantial number of studies related to the interpretation of entrepreneurial behavior. Among these, the Theory of Planned Behavior (TPB) holds a preeminent position [12, 13]. There is a consensus in the literature regarding the validity of intentional models for predicting the entrepreneurial drive, as intentions are understood as antecedents of actual behavior [14]. In order to enhance the college graduates' entrepreneurial behaviors after returning to hometowns, the paramount objective revolves around identifying the variables that exert influence—directly or indirectly—over the entrepreneurial intentions of these graduates." (See Lines 72-80)

**Point 4:** In the introduction, it is very worthwhile to introduce readers in more detail to the social and cultural context in which the university student finds himself.

**Response 4:** Thanks for your suggestion. Indeed, the description of the students' social and cultural context were neglected in the original manuscript. We have now made the following revisions.

"The questionnaires for this study were distributed to 1363 college graduates, who were selected using the convenient sampling method from nine different types of colleges and universities. These nine universities are situated in the western, central, and eastern regions of China, which further enhances the credibility of the research results. These students encompass graduates from different academic backgrounds, such as junior college, undergraduate, and graduate studies. Given that employment choices hold greater significance for these graduates, they are likely to contemplate whether to return to their hometowns for entrepreneurial endeavors." (See Lines 93-100)

**Point 5:** Where are the contributions of this paper? A clear and focused reference to the innovation and uniqueness of the current study is needed in light of the large number of studies already done in this field.

**Response 5:** We are grateful for your commens and suggestions about this. As required, the revision are as follows.

" The contribution of this study is as follows. Returning to Hometown for (RHF) entrepreneurship is the focus of our study. The ongoing decline of rural areas caused by urbanization was the source of this problem. While numerous domestic and foreign investigations have concentrated on college students' entrepreneurial intentions and behaviors, there have been fewer studies centered around 'Returning to Hometown For' (RHF) entrepreneurship. Furthermore, the influencing mechanism of subjective norm, attitude and entrepreneurial self-efficacy to intention for RHF entrepreneurship has been verified by a considerable number of studies. In addition to considering that aspect, this study incorporates the Theory of Man-Land Relationship into TPB. From the perspective of environmental psychology, this study has modified the TPB model by incorporating two factors including place attachment and self-efficacy, and employed the TPB model in interpreting college graduates’ hometown-based entrepreneurial intention." (See Lines 101-112)

**Point 6:** I require that you add text for each one of your hypotheses and justify each one of them by separate and in a better manner you do now. Please try you separate the particular text that corresponds with one or another hypothesis.

**Response 6:** Thanks for your advices about this part. We have revised it in alignment with your suggestion, which made the hypotheses clearer and more readable.

**Point 7:** A very important intervening variable is missing- perceived behavioral control. Please explain.

**Response 7:** We are grateful for the reviewer's suggestion about this. "Perceived behavioral control" is a concept in TPB. Some studies have compared the components of self-efficacy and perceived behacorial control, and noted that self-efficacy may be a more suitable predictor of intention and behavior. To clarify this point, we have included an explanation in the text of hypothesis 3 in Lines 159-170.

**Point 8:** In the methodology, the authors did not properly present the process of using "face-to-face interviews" as a tool for gathering the information - how was it done, who were the interviewers, what was their training process, how was the interviewer's reliability tested, what were the guiding questions? That is, much more details are required than have been presented.

**Response 8:** We appreciate the reviewer's suggestion. The following revisions have been made to the manuscript.

" The questionnaires for this study were distributed to 1363 college graduates during October 2020 to December 2020. They were selected by employing the convenient sampling method from nine different types of colleges and universities. These nine universities are situated in the western, central, and eastern regions of China, which further enhances the credibility of the research results. These students encompass graduates from different academic backgrounds, such as junior college, undergraduate, and graduate studies. Given that employment choices hold greater significance for these graduates, they are likely to contemplate whether to return to their hometowns for entrepreneurial endeavors. The questionnaires are adapted from the mature scale. Finally, 1151 valid questionnaires were obtained, with a valid rate of 84.45%." (See Lines 244-253)

**Point 9:** An article must be self-contained, i.e. it must contain all the information necessary for its comprehension. How the sample has been chosen and to demonstrate that it is representative of the society being studied. I would also recommend adding a table with accurate information about the technical specifications of the study and another table with the comparative demographics between the population and sample.

**Response 9:** We are grateful for your comments and suggestions about this. Regarding the first point about the description of sampling, we have made the following revision.

" The questionnaires for this study were distributed to 1363 college graduates during October 2020 to December 2020. They were selected by employing the convenient sampling method from nine different types of colleges and universities. These nine universities are situated in the western, central, and eastern regions of China, which further enhances the credibility of the research results. These students encompass graduates from different academic backgrounds, such as junior college, undergraduate, and graduate studies. Given that employment choices hold greater significance for these graduates, they are likely to contemplate whether to return to their hometowns for entrepreneurial endeavors. The questionnaires are adapted from the mature scale. Finally, 1151 valid questionnaires were obtained, with a valid rate of 84.45%. " (See Lines 244-253)

As for the second point about adding a table with accurate information about the technical specifications of the study, we have added fit criteria in Table 1, as follows.

**Table 1. Measurement Model Fitting Results.**

| **Latent variables** | **X2/df** | **CFI** | **GFI** | **IFI** | **NFI** | **RMSEA** |
| --- | --- | --- | --- | --- | --- | --- |
| Fit criteria | <4.000 | >0.900 | >0.900 | >0.900 | >0.900 | <0.08 |
| PA | 2.851 | 0.921 | 0.894 | 0.937 | 0.960 | 0.05 |
| ESE | 2.970 | 0.915 | 0.906 | 0.933 | 0.901 | 0.09 |
| SHE | 2.762 | 0.915 | 0.925 | 0.913 | 0.919 | 0.074 |
| AHE | 2.684 | 0.902 | 0.914 | 0.937 | 0.925 | 0.055 |
| HEI | 2.344 | 0.912 | 0.935 | 0.917 | 0.915 | 0.058 |

Concerning the last point regarding the addition of another table, we apologize for the difficulty in comparing the population and the sample. This is beacause that accurate and comprehensive population information cannot be obtained due to the limited access to information.

**Point 10:** Please add the validity and reliability of the measurement instruments. It is not enough to report only the Cronbach's alpha coefficient.

**Response 10:** Thanks for your comments and suggestions for this. In fact, apart from the Cronbach's alpha coefficient, we have reported the validity of the measurement intruments. However, we recognized the need to readjust the structure of the article. As a result, the section pertaining to the measurement instrument validity has now been appropriately repositioned within the Method section.

**Point 11:** In addition, I would recommend adding a table, that shows a list of items used to measure each of the hypotheses.

**Response 11:** We are grateful for your comments and suggestions about this. Based on your feedback, we have inceased the representative items for each variable included in the hypotheses. The details are as follows.

" For example, 'after leaving, my hometown is still an important place in my life', 'a sense of intimacy cannot be found in the other place other than my hometown', 'I am familiar with most parts of my hometown', and 'I rely on my hometown'." (See Lines 276-278)

" For example, 'returning to hometown for entrepreneurship is my career goal', and 'if the job is not deal, I will return to hometown for entrepreneurship'." (See Lines 293-294)

"

**Point 12:** In section 3 it would be better if the control variables used were explained in developing the hypothesis. Where are the results for the control variables used?

**Response 12:** Thanks for your comments and suggestions. As required, we have made the following revisions. The term 'Control variables' has been deleted from the 'measures' section. Although control variables are difficult to explain in the hypotheses, we have further discussed them in the results section. We greatly appreciate your input, and in light of this, we are contemplating incorporating an explanation of the control variables when formulating hypotheses in our future studies.

" This study found that demographic variables such as age and education background can influence entrepreneurial attitude and self-efficacy. This finding aligns with a previous study that indicated college graduates with higher ages and more extensive educational backgrounds are more adaptable [56]. The author suggests that as college graduates' age and educational background increase, so does their personal capability, leading to a more open career concept. Fueled by social and family responsibilities, individuals may increasingly seek self-realization through entrepreneurial behaviors and plans. Additionally, our research identifies gender as a factor influencing entrepreneurial attitudes, consistent with prior studies [57]. One plausible interpretation is that in China, individuals of different genders often assume distinct social responsibilities. " (See Lines 498-507)

**Point 13:** The use of SEM should be able to test the models that have been developed. Unfortunately, how the initial model and the final model after testing is not fully shown. It would be better if this could be done so that the placement of variables or the constructs that build them could be analyzed. Please show the variables with their dimensions.

**Response 13:** We are grateful for your comments and suggestions for this. Upon conducting testing, we have observed differences between the final model and the initial model. Specifically, in section 4, we discovered that H5 has not been verified. This indicates that the attitude towards RHF entrepreneurship had no statistically significant impact on entrepreneurial self-efficacy. As a result, we have depicted the path from attitude to entrepreneurial self-efficacy using a dotted line. As required, we have included the variables along with their respective dimensions. The aforementioned revisions are outlined below.

**Point 14:** The conclusion and discussion section lack significant theoretical reference to the findings. The research should relate to/correspond with theories of entrepreneurship, to the Theory of Planned Behaviour.

**Response 14:** We greatly appreciate your comments and suggestions regarding the conclusion and discussion sections. As required, we have made comprehensive adjustments to this portion. For instance, we have enhanced the section's readability, enriched the theoretical references related to the findings, and expanded the discussion on two theories. You can find detailed information in lines 448 to 563.
