# Supplementary material for: Exploring the impact of college graduates’ place attachment on entrepreneurial intention upon returning to hometowns: A study based on the theory of planned behavior
Source: PLoS One. 2024 Mar 29;19(3):e0300312. doi: 10.1371/journal.pone.0300312 (PMC10980218; doi:10.1371/journal.pone.0300312)
Supplement: Attachment — Submitted filename: Response to Reviewer 4 Comments.docx [file pone.0300312.s002.docx]

Response to Reviewer 4 Comments

**Point 1:** Please emphasize and elucidate the significance of your hypotheses in advancing the understanding of the mediating role of self-efficacy. Based on the literature, it is suggested that self-efficacy positively influences outcomes. We hypothesize that self-efficacy will exert an indirect positive effect between X and Y. Kindly provide further elaboration.

**Response 1:** We sincerely appreciate the reviewer's suggestion. To enhance the clarity and robustness of our study on the mediating role of self-efficacy, we have incorporated essential references and refined the internal coherence of our argument. The revised content is detailed below.

" As established in empirical research, both subjective norm [20] and attitude [21] wield significant influence on the intention for RHF entrepreneurship. Building upon this foundation and our hypotheses, we recognize the pivotal impact that place attachment may exert on the intention for RHF entrepreneurship. Additionally, prior studies has underscored the crucial role of self-efficacy as a predictor of behavior and the strongest determinant of behavioral intention [24,40]. Entrepreneurial self-efficacy, in particular, has been identified as a significant influencer of entrepreneurial intention [41].

Moreover, our review of existing literature corroborates the following points:

Entrepreneurship education can shape entrepreneurial self-efficacy through attitude [41]. Based on this finding, we propose the following hypothesis:

H5: Self-efficacy has a mediating effect on the relationship between attitude towards RHF entrepreneurship and intention for RHF entrepreneurship.

Secondly, place attachment has a significantly positive influence on entrepreneurial self-efficacy [42]. Accordingly, we posit the following hypothesis:

H6: Self-efficacy has a mediating effect on the relationship between place attachment and intention for RHF entrepreneurship.

Finally, subjective norm has a significantly positive influence on self-efficacy [43]. Building upon this observation, we present the following hypothesis:

H7: Self-efficacy has a mediating effect on the relationship between subject norm for RHF entrepreneurship and intention for RHF entrepreneurship.

Drawing from these analyses, we establish multiple mediating relationships with entrepreneurial self-efficacy serving as the mediating variable within the TPB model. Subsequently, we have refined the model for the third iteration, and the updated theoretical model diagram is depicted in Fig 1." (See Lines 218-241)

**Point 2:** Additionally, please offer more insights and details on the demographics of the respondents. Present this information in tabular form.

**Response 2:** We greatly appreciate the reviewer’s comments and suggestions. As required, we have added more details of the demographics of the respondents and presented this information in tabular form. The detailed revisions can be found in the specified lines. (See Lines 259-260)

**Table 1. The Demographics of the Respondents.**

| **Variables** | **Level** | **Quantity** | **Ratio** |
| --- | --- | --- | --- |
| Gender | Male | 696 | 60.47% |
|  | Female | 455 | 39.53% |
| Academic backgrounds | Associate degree or below | 437 | 37.97% |
|  | Bachelor’s degree | 468 | 40.66% |
|  | Master’s degree or above | 246 | 21.37% |
| Professional backgrounds | Humanistic and social science | 582 | 45.94% |
|  | Science and Engineering | 685 | 54.06% |
| Entrepreneurial backgrounds | Yes | 143 | 11.29% |
|  | No | 1124 | 88.71% |

**Point 3:** Separate implications into theoretical and managerial categories.

**Response 3:** We are grateful for the reviewer’s suggestions. We have modified the structure of the Implications section and split it into theoretical and managerial categories, which can be seen from lines 579 to lines 634.

**Point 4:** Proofreading is required for clarity, as some sentences are difficult to understand and contain grammatical issues. This version aims to enhance clarity and flow, making it easier for readers to grasp the intended meaning.

**Response 4:** Thanks for your valuable suggestion. As required, we have re-examined the manuscript and modified the problematic statement. These changes are clearly delineated in the manuscript using the track changes feature. We hope that this version can make it easier for readers to grasp the intended meaning.
